# Editorial: Women in drug metabolism and transport: 2021

**DOI:** 10.3389/fphar.2022.966797

**Published:** 2022-08-04

**Authors:** Claudia Bregonzio, Sara Eyal, Franciska Erdő, Mariela Fernanda Pérez

**Affiliations:** ^1^ Departamento de Farmacología Otto Orsingher, Facultad de Ciencias Químicas, Instituto de Farmacología Experimental Córdoba (IFEC-CONICET), Universidad Nacional de Córdoba, Córdoba, Argentina; ^2^ Hebrew University of Jerusalem, Jerusalem, Israel; ^3^ Pázmány Péter Catholic University, Budapest, Hungary

**Keywords:** bioavailability, ADME & toxicity, pharmacokinetic, physiologically-based pharmacokinetic, biosimilar agents

Pharmacokinetic (PK) studies during drug development are crucial in determining standard dose regimens, enabling rational dose adjustment and attempts to ensure safety during clinical use, having as much relevance as pharmacodynamics aspects. This special issue includes very important findings performed by women in this relevant area of science. One of the presented manuscripts also directly applies to populations of female patients (e.g., the major target population for denosumab).

Physiologically-based pharmacokinetic (PBPK) modeling has become increasingly widespread within the pharmaceutical industry over the last decade. PBPK modeling is a simulation approach with several applications, accepted by regulatory agencies primarily to evaluate enzyme-based drug-drug interactions (Grimstein et al., 2019). PBPK can be applied to evaluate changes in absorption, metabolism, distribution, and elimination (ADME). The prediction of pharmacokinetics in a cohort of virtual patients is made using in vitro-in vivo extrapolation techniques. Montanha et al., performed an interesting study using PBPK modeling to predict the effect of different stages of liver disease on the pharmacokinetics of dexamethasone in COVID-19 patients.

The use of innovative tools for determining drug metabolism and transport as well as the in vitro-to-in vivo extrapolations are critical during preclinical and clinical studies. In this sense, elucidation of metabolites and the metabolic pathways for new drugs is an important stage of the human ADME studies required to corroborate whether these may cause pharmacological or toxicological effects. In the context of early clinical drug development, the accelerator mass spectrometry has been applied to assess various drug characteristics including but not limited to PK, mass balance, absolute bioavailability, and metabolite profiling using a microdose/microtracer approach. Such integrative approach accelerates clinical development (Muehlan et al., 2018). Huynh et al. combine this innovative method with *in vitro* studies in order to characterize ADME in humans with the use of rat samples for metabolite structure elucidation of major human metabolites, and to identify human enzymes involved in the metabolism of an orally available, promising new drug with proven efficacy in preclinical animal models of multiple sclerosis (Pouzol et al., 2021a) and acute lung injury (Pouzol et al., 2021b), and favorable clinical profile following single and multiple-dose (Huynh et al., 2021a; Huynh et al., 2021b) administration. Results from their *in vitro* and *in vivo* studies highlight the relevance of preclinical investigations in supporting the identification of metabolites in humans, allowing determining potential successive clinical studies.

Another determining factor in the clinical response to medications are polymorphisms in genes related to their pharmacokinetics or pharmacodynamics because such polymorphisms can alter the protein function. Examples include genes encoding isozymes of cytochrome P450 (CYP) or uridine 5′-diphospho-glucuronosyltransferases (UGTs), organic anion transporting polypeptides (OATPs), and drug receptors. Barliana et al. summarize the data from human studies published in the last 10 years regarding gene polymorphisms that influence the response to systemic lupus erythematosus therapy. The authors propose personalized medicine to provide individual therapy based on genetic profiles to obtain effective treatments for autoimmune diseases.

Comparative PK studies designed to document similar PK profiles for key parameters of biosimilar and reference medicinal products are an essential component of biosimilar development programs. Biosimilars can improve the health of patients by increasing their accessibility to biological molecules due to decreasing healthcare-associated costs (Mulcahy et al., 2018). Denosumab is a fully human IgG κ-type monoclonal antibody, useful to increase bone mass in patients who are at high risk of fractures. Its biosimilars are being actively developed worldwide (Zhang et al., 2020; Jose et al., 2021; Zhang et al., 2021). However, no denosumab biosimilar has been marketed in China to date. Chen et al., phase I clinical study took the lead and showed that the PK, pharmacodynamic and anti-drug antibody profiles of the denosumab biosimilar (CMAB807) were similar to those of denosumab. Furthermore, the safety data were also comparable between the biosimilar and the parent drug. These results support the efficacy of CMAB807 as a denosumab biosimilar.

PK preclinical studies in the context of drug development for addiction treatment is extremely relevant since many natural-derived drugs are used in informal self-help networks and treatment centers worldwide, even without license. The abuse of psychostimulants, opioids and alcohol among other substances is considered a worldwide flagellum being the maintenance of the individual abstinence period the major challenge for the addiction treatments. In this sense, Martins et al., performed an interesting study showing the role of drug transporters in the bioavailability of ibogaine (psychoactive indole alkaloid), used as an oral treatment for substance use disorders (although being unlicensed in most countries). The results obtained by these authors are highly relevant since several preclinical and clinical studies have described long-term drug withdrawal from various substances, including opioids, alcohol, and psychostimulants, and sustained reductions in withdrawal-related-depressive symptoms after ibogaine administration.

Continuous learning about different mechanisms involved in the distribution, metabolism, excretion and the role of several diseases affecting them are extremely necessary in the clinic for better decision making. Moreover, new information regarding compounds with high potential therapeutic aspects becomes relevant in an actively changing and demanding society.
